# A Case of Wilson's Disease With Early Neuropsychiatric and Late Hepatic Manifestations

**DOI:** 10.7759/cureus.90122

**Published:** 2025-08-14

**Authors:** An Phuc D Ta, Jiahua Zhou, Megan D Hsu, Harrison G Chu, Ashlyn Huynh, Gary Chu

**Affiliations:** 1 College of Medicine, California Northstate University, Elk Grove, USA; 2 Health Service Administration, American River College, Sacramento, USA

**Keywords:** early psychiatry late hepatic wilson's disease, neuropsychiatric hepatic wilson, untreated wilson's disease, wilson, wilson disease, wilson's, wilson's disease, wilson's disease hepatic, wilson's disease neuropsychiatric manifestation, wilson's disease psychiatric

## Abstract

Wilson’s disease (WD) is a rare genetic disorder that affects copper metabolism, primarily presenting with hepatic and central nervous system symptoms. Early detection can be challenging due to its variable presentation. In this case, we discuss a 44-year-old female with a six-year history of chronic neurogenic pain, migraines, alcohol use disorder, major depressive disorder, and recent unexplained abdominal pain. She presented to the emergency room with ascites, fulminant hepatic failure, and acute kidney injury. Despite her previously stable health, her condition rapidly decompensated over a month with the onset of hepatic failure. Decompensated cirrhosis secondary to non-alcoholic steatohepatitis or alcoholic hepatitis was suspected. Still, a positive ceruloplasmin study and the presence of Kayser-Fleischer rings observed on slit-lamp examination ruled in WD. This case highlights a late-onset presentation of WD. With early diagnosis and treatment, the fatal sequelae of WD can be prevented. Along with this case, the classifications of WD, its common and unique features, and the diagnostic criteria were reviewed.

## Introduction

Wilson’s disease (WD) is a disorder resulting in the accumulation of copper that causes organ damage to the liver, brain, kidneys, eyes, heart, joints, and other organs [1]. It is difficult to diagnose and often misdiagnosed in part due to its low prevalence of approximately 1:45000 in the general population [1]. This disease is characterized by impaired copper metabolism due to a homozygous or heterozygous autosomal recessive mutation of the ATP7B gene (13q14.3). Multisystem organ damage from copper accumulation and oxidative toxicity primarily affects tissues with local ATP7B expression, such as the brain, liver, kidneys, and eyes [1,2]. WD affects 1 in 30,000 individuals with a carrier frequency of one in every 90 and does not show gender proclivity [3]. The onset age for WD typically ranges from four to 40 years, although cases have been reported from as early as eight months old with transaminitis [4] to as late as 75 years old with new onset of Parkinsonism [5]. The average age at diagnosis is generally between 18.5 ± 11 years and 28 ± 9 years [6].

From infancy to puberty, WD often presents with gastrointestinal or hemolytic symptoms [7-11]. From puberty onwards, neurological symptoms become more prevalent, starting between ages 15-21 or 20-30 years [9,11,12]. Disease onset after the age of 40 is considered a late manifestation, with multiple clinical patterns documented in the literature. These are (1) hepatic predominant with mild symptoms, (2) isolated Kayser-Fleischer ring (KFR), and (3) neurological predominant, with or without liver involvement [13]. The neurological subset was found to be more common in this age group [6].

The classic diagnostic triad of WD includes low ceruloplasmin, high 24-hour urine copper, and KFR. The Leipzig score incorporates this triad along with other findings to establish a diagnosis. The European Association for the Study of the Liver and the European Society for Paediatric Gastroenterology, Hepatology, and Nutrition support the use of the Leipzig score. In contrast, the Indian Association uses a modified Leipzig score [9,14,15]. The difference between the Leipzig and modified Leipzig scores is that the latter adds a point for a positive family history and very low ceruloplasmin levels (<5 mg/dL), removes the D-penicillamine challenge test, utilizes MRI findings and neurobehavioral symptoms, and puts less weight on invasive tests, including liver biopsy. The American Association for the Study of Liver Diseases recommends a stepwise algorithmic approach, with a detailed medical and family history of liver disease or early-onset neuropsychiatric disease, slit lamp exam, 24-hour urinary copper excretion, and serum ceruloplasmin level, followed by hepatic copper quantification and histological assay. Finally, molecular testing for individuals in whom the diagnosis is difficult to establish but remains a consideration is recommended [16]. All guidelines above agree that first-degree relatives of the patient should also be screened.

Imaging can aid in diagnosing neurological WD and contribute to a positive Leipzig score. A T2-weighted brain MRI can show hyperintensity in the putamen, globus pallidus, internal capsule, and thalamus. Atrophy in the caudate nucleus, brainstem, and cerebral and cerebellar hemispheres can also be seen. Notable radiological signs on T2-weighted MRI images seen in a minority of patients include the "face of the giant panda" in the midbrain and "panda cub" in the pons [17,18]. The "face of the giant panda" is a pattern of hyperintensities in the midbrain tegmentum surrounding the red nucleus and substantia nigra. Meanwhile, the "panda cub" sign is a hypointense medial longitudinal fasciculus and central tegmental tract with a hyperintense aqueduct. Still, these are non-specific signs, as they can also be observed in unrelated diseases such as Leigh syndrome. Lastly, sleep studies can demonstrate signs and symptoms associated with neurological WD, including cataplexy, REM behavioral disturbances, and restless leg syndrome [19].

When diagnosed, WD can be effectively treated with copper-chelating medications such as penicillamine or trientine and oral zinc. If the liver is severely damaged, patients can be candidates for a liver transplant, which is the definitive curative treatment for WD. Liver transplants have a survival rate of 87% at 15 years and may also have therapeutic effects on neurological deficits as well [20].

Previous case studies have shown that the late-onset type of WD often presents with predominantly neuropsychiatric symptoms [21,22]. Here, we report a case of WD in a middle-aged female with a neuropsychiatric history but without a history of prior hepatic disease. We accompany this with a brief review of subtypes of WD and possible presentations.

## Case presentation

**Figure 1:**
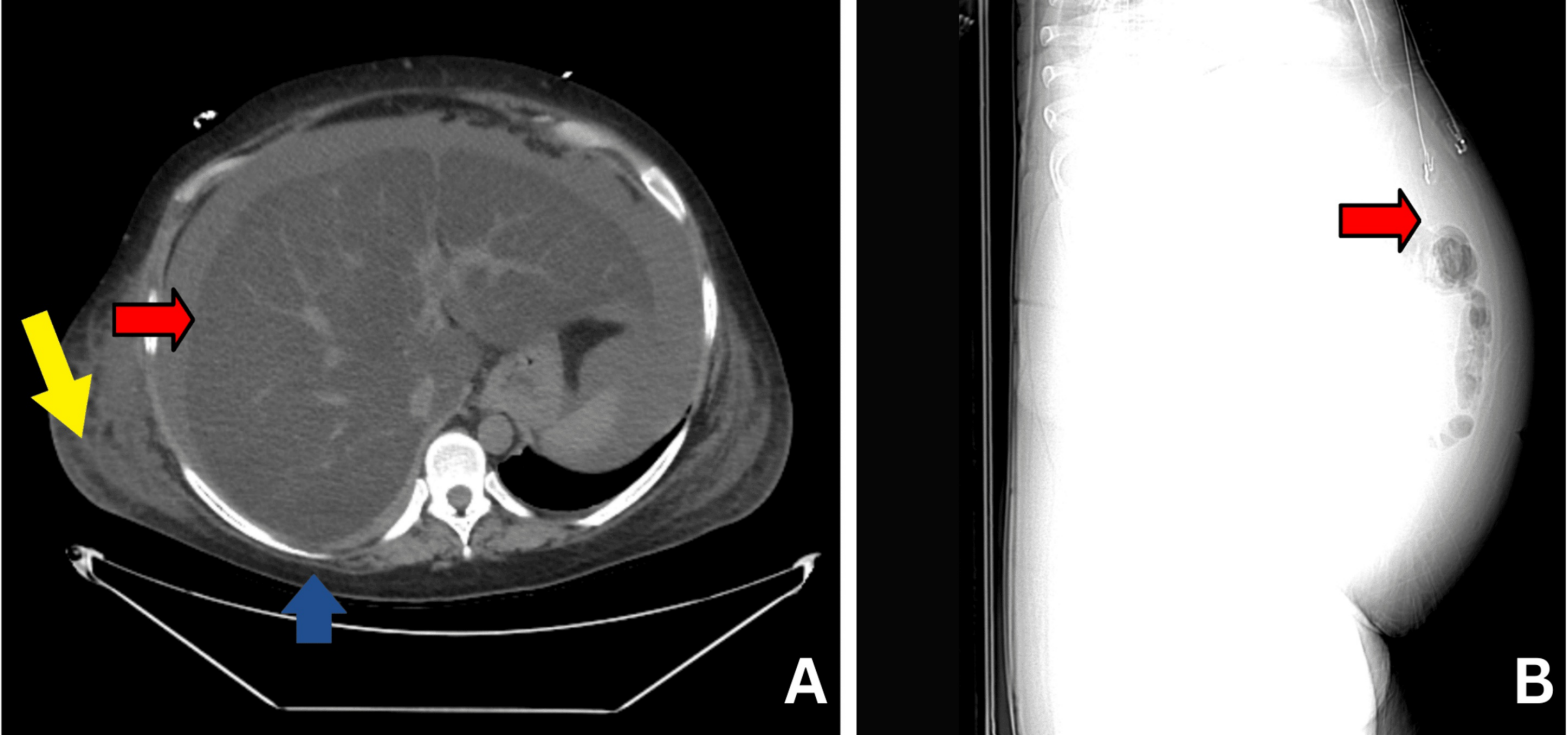
Day 1 imaging of WD hepatic manifestations (A) Day 1 ED non-contrast CT abdomen pelvis shows moderate ascites (red arrow), fatty liver, no free air or abscess, mild diffuse anasarca (yellow arrow), and atelectasis (blue arrow) in the inferior right lobe. (B) Day 1 CT abdomen pelvis in lateral view showing abdominal shadow representing ascites. WD: Wilson’s disease, ED: emergency department, CT: computed tomography

A 44-year-old female presented to the emergency department (ED) in 2023 for progressively worsening diffuse abdominal pain with distention and bilateral leg swelling. She had a past medical history of migraines, bilateral hand numbness (2015), major depressive disorder (2016), foot numbness (2018), plantar fasciitis, possible peripheral neuropathy with a negative nerve conduction study (2019), and left flank pain (2020). Three months ago, she experienced fatigue, abdominal pain, and abdominal distension. An abdominal CT scan showed fatty liver with moderate ascites and a normal spleen (Figure 1A). A colonoscopy two weeks earlier revealed diverticulosis and a small hemorrhoid. She was treated empirically for diverticulitis with a course of antibiotics, but continued to have abdominal pain. Her ED chief complaint was a new onset of bilateral leg and abdominal swelling. She had no significant family history and denied recent alcohol usage or previous history of liver disease, infections, or drug use.

Upon physical examination, she was afebrile with vital signs showing a blood pressure of 98/66 mmHg, a pulse of 114 beats per minute, a respiratory rate of 20 breaths per minute, and oxygen saturation of 98% on room air. Her abdomen was peritonitic, diffusely tender with guarding and distention. She exhibited 3+ pitting edema in her thighs. S1 and S2 heart sounds were heard without murmur, and there was no respiratory distress or abnormal breath sounds upon pulmonary examination. No scleral icterus was noted, and her skin was warm without signs of jaundice. She was alert and oriented to place, person, and time.

**Figure 2:**
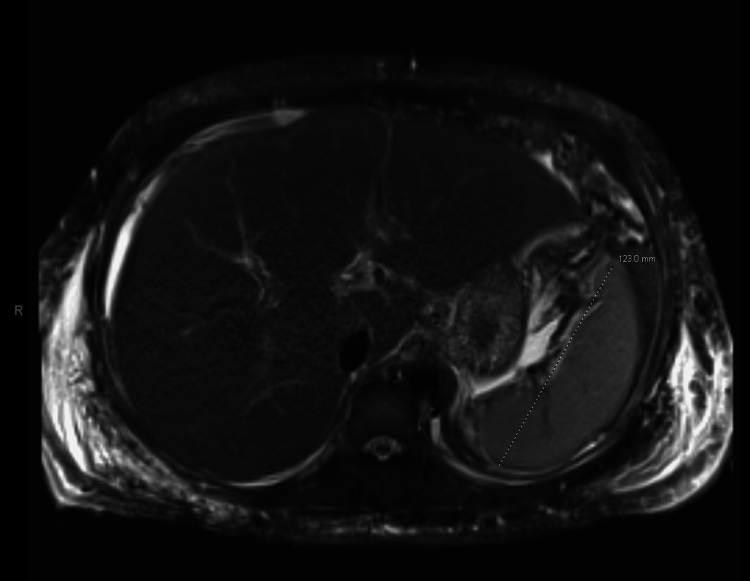
MRI abdomen on day 8 of hepatic manifestations Imaging shows severe hepatic steatosis, moderate volume ascites, possible cirrhosis, and a normal spleen size of 12.3 cm. MRI: magnetic resonance imaging

Imaging included a non-contrast CT abdomen on day 1 and an MRI of the abdomen on day 8. A non-contrast CT abdomen revealed atelectasis in the inferior right lower lobe, moderate ascites, no free air or abscess, and mild diffuse anasarca (Figure 1). MRI of the abdomen on day 8 showed severe hepatic steatosis, mild undulation of the liver contour, moderate volume ascites, nonspecific borderline enlarged periportal lymph nodes, small bilateral pleural effusions with patchy atelectasis at the right lung base, and diffuse body wall edema (Figure 2). Ascitic fluid analysis showed a serum ascites albumin gradient score of 1.4, indicative of portal hypertension; serum protein loss at 1.4, consistent with hepatic cirrhosis; and no evidence of spontaneous bacterial peritonitis. Esophagogastroduodenoscopy was negative for varices. These findings, in conjunction with her significant obesity, led to a diagnosis of an acute decompensated cirrhosis event secondary to NASH and AKI.

Laboratory tests included complete blood count (CBC), complete metabolic panel (CMP), liver function tests (LFTs), kidney function tests, ceruloplasmin, and urinary copper. CBC revealed leukocytosis from days 1 to 11, low red blood cell (RBC), hemoglobin (HGB), and hematocrit (HCT) from days 1 to 11, and high mean corpuscular volume (MCV) from days 1 to 11 (Table 1). CMP indicated hyponatremia from days 1 to 4, hypokalemia on day 10, and hypochloremia from days 1 to 5. LFTs showed elevated aspartate aminotransferase (AST) (AST/ALT >2), alkaline phosphatase (ALP), and albumin on days 1, 2, and 5, as well as elevated total and direct bilirubin on days 1, 2, 5, and 9 (Table 1). On day 5 of hospitalization, she was worked up for potential WD and had a low ceruloplasmin level of 15. On day 10, she had a normal 24-hour urinary copper level of 22 mcg. At this point, WD was considered, but a definitive diagnosis could not be made. The patient was discharged for outpatient workup of their conditions, with WD in consideration.

**Table 1:** Hospitalization lab values from admission (day 1) to discharge (day 11) The patient had hyponatremia on days 1-4, hypokalemia on day 10, hypochloremia on days 1-5, hypocapnia on days 1-9, high blood urea nitrogen on day 3 and low on days 10-11, high creatinine on days 1-8, high AST on days 1, 2, and 5, high ALKP on days 1, 2, and 5, high total bilirubin on days 1, 2, 5, and 9, high direct bilirubin on days 1, 2, 5, and 9, high albumin on days 1, 2, and 5, elevated WBC days on 1-11, low RBC, HGB, and HCT on days 1-11, and high MCV on days 1-11. NA: sodium, K: potassium, Cl: chloride, CO₂: carbon dioxide, BUN: blood urea nitrogen, ALT: alanine aminotransferase, AST: aspartate aminotransferase, ALKP: alkaline phosphatase, TBILI: total bilirubin, BILID: direct bilirubin, ALB: albumin, WBC: white blood cell count, RBC: red blood cell count, HGB: hemoglobin, HCT: hematocrit, MCV: mean corpuscular volume, NRBC: nucleated red blood cells, N: normal range, mEq/L: milliequivalents per liter, mg/dL: milligrams per deciliter, U/L: units per liter, g/dL: grams per deciliter, K/uL: thousands per microliter, M/uL: millions per microliter, fL: femtoliters, %: percent

	Day 1	Day 2	Day 3	Day 4	Day 5	Day 6	Day 7	Day 8	Day 9	Day 10	Day 11
NA (N: 135-145 mEq/L)	125	128	130	129	132	135	138	138	139	140	139
K (N: 3.5-5.3 mEq/L)	4.3	4	4	4.1	4	3.8	3.7	3.3	3.5	3.2	3.6
Cl (100-111 mEq/L)	92	96	98	98	99	100	103	103	103	103	102
CO2 (24-33 mEq/L)	22	23	22	20	21	22	23	22	23	24	25
BUN (N: 7-27 mg/dL)	27	27	28	27	25	20	16	11	8	5	4
Creatinine (N: <1.11 mg/dL)	3.21	3.16	3.01	2.61	2.23	1.92	1.45	1.2	0.98	0.82	0.83
ALT (N: 0-41 U/L)	17	13			10				8		
AST (N: 10-40 U/L)	56	43			42				34		
ALKP (N: 37-117 U/L)	278	208			143				80		
TBILI (N: 0.2-1.2 mg/dL)	3.4	2.7			2.6				2.1		
BILID (N: 0-0.3 mg/dL)	2	1.7			1.5				1.1		
ALB (N: 4-5 g/dL)	2.3	2.1			3.6						
WBC (N: 3.5 - 12.5 K/uL)	22.8	14.3	15.1	13.8	14.5	14	13.6	13.2	14.6	14.3	13
RBC (N: 3.6 - 5.10 M/uL)	2.94	2.51	2.58	2.33	2.26	2.17	2.17	2.16	2.29	2.21	2.24
HGB (N: 11-15 g/dL)	10.4	8.6	8.9	8.3	7.8	7.6	7.5	7.4	7.9	7	7.6
HCT (N: 34-46%)	31.1	25.7	26.8	24.5	23.5	23	22.6	22.1	23.7	22.9	23.5
MCV (N: 80-100 fL)	106	102	104	105	104	106	104	102	104	104	105
NRBC (N: 0)	0	0	0	0	0	0	0	0	0	0	0

Post-discharge, KFR rings were seen on a slit-lamp exam by an ophthalmologist. A week later, she returned to the ED with altered mental status and was admitted to the ICU for hepatic encephalopathy, severe sepsis, and acute kidney injury. She passed away on day 9 due to decompensation. Post-mortem surgical liver biopsy results showed an elevated hepatic copper concentration of 447.3 ug/g (N:15.0-55.0-/55.0 μg/g), confirming a diagnosis of WD.

## Discussion

This case involves a patient with WD who first experienced neurological symptoms, including migraines and bilateral hand numbness (2015), foot numbness (2018), plantar fasciitis, and possible peripheral neuropathy (2019). These were followed by left flank pain (2020). Given the rarity of WD and the broad range of differentials, a primary liver pathology as the cause of neuropsychiatric disturbances was not initially considered. Other considerations, such as major depressive disorder, are higher on the differential list, especially as a comorbidity. As mentioned previously, the clinical prevalence of WD is low, with another source estimating between 1:30,000 and 1:50,000 [3]. Consequently, many general practitioners, pediatricians, and neurologists may never encounter a WD patient, leading to misdiagnosis or delayed diagnosis. While personality changes alongside liver pathology are easily recognizable as WD in theory, cases with neuropsychiatric onset can present vaguely and go unnoticed until more severe hepatic damage occurs. Neurological WD was found to be missed in two-thirds of initial presentations in one study, resulting in an average 13-month delay in diagnosis [23].

A previous case reported a 17-year-old male who developed intermittent paresthesias and weakness in his hands and feet at least six months before showing more typical WD symptoms [22]. Electrophysiological and pathological studies suggested a mixed-type neuropathy, similar to our patient, who had bilateral hand paresthesias despite a nerve conduction study without evidence of axonal or demyelinating sensory neuropathy, entrapment neuropathy, or other abnormalities. Ala et al. described two siblings: a 72-year-old woman with progressive neurological disability followed by sub-fulminant liver failure and her 70-year-old brother, who had a mild hand tremor and depressive disorder from the age of 45 [21]. His liver biopsy revealed steatosis, minimal fibrosis, and elevated hepatic copper content. These findings are similar to our patient’s history of depressive disorder and unremarkable liver imaging, indicative of only steatosis prior to the development of ascites and fulminant liver failure.

The Leipzig classification divides WD into three subtypes: neurological, hepatic, and others [24]. Patients with neuropsychiatric symptoms at diagnosis fall under the neurological subtype, which can be further divided based on the presence of liver disease. The hepatic subtype is defined by biochemical evidence of liver disease and can be subdivided by chronicity and severity. The other subtype is diagnosed in the absence of both neurological symptoms and liver disease. Each subtype has a spectrum of presentations, varying in age of onset, demographics, symptoms, and treatment response, even among twin pairs. This variability is proposed to be due to differences in genotypic penetrance, mutation, and environmental influences such as diet, exercise, and stress via epigenetic modifications [25].

The most common extrahepatic site of copper accumulation is the central nervous system. Neurological and psychiatric symptoms can occur in isolation but are often paired together. Up to 20% of WD patients initially present with only psychiatric features, one-third predominantly with psychiatric symptoms, and two-thirds eventually develop psychiatric symptoms [26]. Behavioral changes are commonly seen in WD, with prevalence ranging from 46% to 71%. There are two possibilities for this presentation: a psychiatric picture with depression, irritability, or anxiety, or neurologically as dystonia and Parkinsonism [26]. The most common initial neurological signs are tremors (rest, action, attitude) and dysarthria [26]. Executive function can be diminished due to involvement of the basal ganglia and subcortex, while verbal intelligence, episodic memory, and visual-spatial abilities are often preserved [26]. The exact mechanism linking brain disease, elevated copper levels, and neuronal dysfunction with ATP7B expression is unclear to date [27-29].

Histologically, these cases of neurological WD commonly show milder hepatic disease with minimal fibrosis, fatty vacuoles, and steatosis, suggesting possible liver resistance or genetic protection against copper toxicity that explains the delayed age of onset [13]. Our case report supports these observations, as this patient experienced vague neuropsychiatric symptoms without evidence of active hepatic diseases years prior to the onset of fulminant liver failure. There are hepatic, neuropsychiatric, and miscellaneous clinical features of WD that resemble a vast pool of other differential diagnoses (Table 2).

**Table 2:** Possible clinical features and diagnoses for a WD case WD has a myriad of presentations that can create complexity in diagnoses [2,13,27-29]. WD: Wilson’s disease, AST: aspartate aminotransferase, ALT: alanine aminotransferase, ALP: alkaline phosphatase, ADHD: attention-deficit/hyperactivity disorder, REM: rapid eye movement

	Clinical patterns	Differentials
Hepatic	Abdominal pain; ascites; dark urine; epistaxis; family history of liver disease; fatty infiltration, contour irregularity associated with cirrhosis and regenerative nodules; fever; grade I–IV on West Haven Classification for hepatic encephalopathy; gynaecomastia; hemolytic anemia with (–) direct Coombs test; hepatosplenomegaly; high AST/ALT ratio; jaundice; low ALP, low ALP ratio; nausea, vomiting; peripheral edema; portal hypertension; pruritus; right-lobe atrophy on ultrasound	Acute hepatitis, acute jaundice, acute liver failure, acute nephritis, alcoholic hepatitis, asymptomatic hepatomegaly, autoimmune hepatitis, compensated/decompensated cirrhosis, hemolytic anemia, hepatic encephalopathy, hepatocellular carcinoma, immunoglobulin M nephropathy, isolated splenomegaly, non-alcoholic fatty liver disease, non-alcoholic steatohepatitis, non-icteric hepatitis, persistent/intermittent elevation of serum aminotransferases, and upper gastrointestinal hemorrhage
Neuropsych	Abnormal gait; acute/chronic psychosis; anxiety; autonomic dysfunction (sympathetic or parasympathetic); cataplexy episodes; changes in personality or behavior (i.e., abnormal aggressiveness, criminal behavior, difficulties at school or work); chorea; deficits in attention, visuospatial perception and reasoning, learning and memory, and verbal and abstract reasoning; depressed mood; drooling; dysarthria; dystonia; insomnia; daytime sleepiness; mania; mild tremor; parkinsonism; polyneuropathy (paresthesias and weakness); status epilepticus; suicidal ideation; motor tics	ADHD, anorexia nervosa, bipolar disorder, bulimia, dementia, major depressive disorder, mild cognitive disorder, neuropathy of mixed type, obsessive-compulsive disorder, partial simple or complex seizure, REM sleep behavior disorder, restless leg syndrome, rheumatic chorea, schizophrenia, subacute sclerosing panencephalitis, Tourette’s syndrome
Others	Abnormal menstruation and infertility, amenorrhea, disorders of growth, galactorrhoea, hypoglycaemia, hypoparathyroidism, hypothyroidism, recurrent abortions, epistaxis, and nephrolithiasis	Genu valgum, juvenile polyarthritis, recurrent fractures, rickets, Fanconi syndrome

## Conclusions

As there are many clinical features involving several organ systems, there is a vast differential list that can cause symptoms in WD. The severe sequelae of this disease are treatable with chelation therapy, but it requires recognition of the signs of WD early on. Clinician education on the recognition of WD is important, as this is a fatal disease when left untreated. Our case reinforces the importance of holding a higher index of suspicion for WD when a patient presents with abdominal pain of hepatic etiology, accompanied by a neuropsychiatric past medical history.
